# Targeted Foliar Micronutrient Modulates Phenolic Accumulation in Olive Leaves

**DOI:** 10.3390/plants15162503

**Published:** 2026-08-19

**Authors:** Mariem Zakraoui, Marija Polić Pasković, Sondes Stambouli-Essassi, Igor Palčić, Smiljana Goreta Ban, Barbara Soldo, Nataly Milovan, Sunčana Simonić-Kocijan, Paula Žurga, Danko Cvitan, Igor Pasković

**Affiliations:** 1 Laboratory of Biodiversity, Biotechnology and Climate Change (LR11ES09), Department of Biology, Faculty of Sciences of Tunis, University of Tunis El Manar, Tunis 1060, Tunisia; mariem.zakraoui@fst.utm.tn (M.Z.); sondes.stambouli@fst.utm.tn (S.S.-E.); 2 Department of Agriculture and Nutrition, Institute of Agriculture and Tourism, Karla Huguesa 8, 52440 Poreč, Croatia; smilja@iptpo.hr (S.G.B.); nataly@iptpo.hr (N.M.); danko@iptpo.hr (D.C.); paskovic@iptpo.hr (I.P.); 3 Department of Chemistry, Faculty of Science, University of Split, R. Boškovića 33, 21000 Split, Croatia; barbara@pmfst.hr; 4 Department of Prosthodontics, Faculty of Dental Medicine, University of Rijeka, Krešimirova 40, 51000 Rijeka, Croatia; suncanask@fdmri.uniri.hr; 5 Teaching Institute of Public Health Primorsko-Goranska County, Krešimirova 52a, 51000 Rijeka, Croatia; paula.zurga@zzjzpgz.hr

**Keywords:** *Olea europaea* L., foliar fertilization, Leccio del Corno, zinc, copper

## Abstract

Olive (*Olea europaea* L.) leaf phenolics are increasingly valued across various industrial sectors. This study evaluated whether foliar application of copper (Cu), zinc (Zn), and manganese (Mn) can influence phenolic accumulation in olive leaves. One-year-old olive plantlets grown under greenhouse conditions were sprayed with these micronutrients. Samples of treated mature leaves were collected at 30, 60, and 90 days after treatment (DAT), whereas young leaves that developed after application were sampled at 90 DAT. Foliar fertilization significantly influenced the phenolic profile of olive leaves depending on the micronutrient applied, the sampling time, and the age of the leaves. The highest mean oleuropein concentration was recorded at 30 DAT (209.43 mg/100 g DW), driven mainly by the Cu × 30 combination (302.36 mg/100 g DW), whereas 90 DAT showed a significant decrease in most phenolic classes. In young leaves, oleuropein concentrations were an order of magnitude higher (2661–5105 mg/100 g DW) than those measured in mature leaves (14.5–302 mg/100 g DW). Furthermore, correlation analysis revealed nutrient-specific modulation of phenolic metabolism: Cu was positively associated with secoiridoids in mature leaves, Zn was positively associated with secoiridoids at 90 DAT, and Mn was negatively associated with flavonoids and with Cu and Zn at 90 DAT in young leaves. These findings suggest that foliar fertilization affects phenolic pathways differently depending on the applied micronutrient and the leaf developmental stage, providing new insights into the temporal regulation of olive leaf phenolic metabolism and identifying suitable harvest periods for the targeted phenolic compounds.

## 1. Introduction

Olive (*Olea europaea* L.) is one of the most emblematic and widely cultivated fruit tree species in the Mediterranean region. Olive cultivation is also a major agricultural sector, contributing significantly to agricultural income and rural development in many Mediterranean countries. Olive trees are well adapted to harsh environmental conditions, making them a key component of sustainable agricultural systems in semi-arid regions. Moreover, olive cultivation is widespread worldwide, with an estimated area of 9 million hectares [1].

In recent years, attention has grown not only for olive oil but also for olive leaves, which are recognized as a rich source of bioactive compounds [2]. These bioactive compounds, particularly oleuropein, flavonoids, and phenolic acids, exhibit important health-promoting properties. They are known for their strong antioxidant capacity and have shown antimicrobial, anticancer, and neuroprotective activities [3,4]. In addition, they exhibit anti-inflammatory, antihypertensive, antidiabetic, and cardioprotective effects [4,5,6]. Moreover, olive leaf phenolics are increasingly valued across various industrial sectors for their natural antioxidant and antimicrobial properties. They are widely used in food preservation, functional foods, cosmetics, and pharmaceutical formulations [3,7].

Copper (Cu), manganese (Mn), and zinc (Zn) are essential micronutrients required for optimal plant growth and metabolic function. These elements serve as enzymatic cofactors in key physiological processes, including photosynthesis, respiration, and the regulation of oxidative stress [8,9,10]. Manganese is an essential cofactor for photosynthesis, particularly in the photolysis of water, and participates in enzymatic activation related to amino acid metabolism [11]. It is also directly implicated in the regulation of secondary metabolism, including the phenylpropanoid pathway responsible for the biosynthesis of phenolic compounds [12,13]. In addition to Mn, other micronutrients, such as Cu and Zn, have been associated with phenolic metabolism due to their roles in enzymatic activation and redox reactions. Copper is primarily involved in chlorophyll production, cellular respiration, and protein synthesis, thereby strengthening plant tissues and their resistance to diseases. Zinc is an essential micronutrient for protein production in plants. Indeed, it is essential for the metabolism of plant hormones by participating in tryptophan synthesis, a prerequisite for auxin formation [11].

Several studies have examined how mineral nutrition and fertilization affect olive physiology and phenolic metabolism, generally reporting positive outcomes. For instance, Tekaya et al. [14] demonstrated that foliar fertilization improved olive tree performance by enhancing key leaf physiological parameters. Similarly, Souza et al. [15] highlighted the fundamental role of mineral nutrients in maintaining normal metabolic functions and showed that nutrient imbalances can lead to significant physiological and biochemical alterations. Abdeljelil et al. [16] showed that foliar fertilization, combined with seasonal variation, significantly affected the phenolic composition of leaves in the Chemlali olive cultivar. In addition, Pasković et al. [17] found that manganese application positively influenced both mineral uptake and phenolic content in olive plantlets. More recently, Vidović et al. [18] confirmed that manganese nutrition modulates the biophenolic profile across different olive tissues. Collectively, these findings indicate that mineral fertilization can significantly influence phenolic metabolism in olive trees, often increasing phenolic compounds.

These findings highlight that foliar micronutrient fertilization can significantly modulate the biosynthesis of phenolic compounds in plants. Olive leaves, although not the primary target of the crop, play a crucial role in the plant’s overall metabolism. Moreover, leaves are the primary source of photoassimilates and metabolites that can be mobilized and transported to developing organs, particularly the fruit [19,20]. Consequently, sugar and phenolic compound levels are likely to indirectly influence fruit development and biochemical composition [21,22,23,24]. Thus, foliar fertilization could strengthen the physiological activity of leaves and their defense mechanisms while promoting better availability of assimilates necessary for the growth and nutritional value of fruits [21,25]. These effects vary depending on several factors, including the type of micronutrient, the application method (foliar or soil), and the stage of leaf development [12,17,26].

Although fertilization is known to influence the phenolic profile of plants [14,16], limited attention has been paid to the comparative effects of other essential micronutrients, such as Cu, Zn, and Mn, or to the combined effects of fertilization and time on the dynamics of phenolic compounds in olive leaves. Such an approach would provide a better understanding of plant responses to micronutrient fertilization and help optimize fertilization strategies to improve leaf biochemical quality. This gap underscores the relevance of the present study, which aims to investigate the impact of foliar fertilization with micronutrients (Zn, Cu, and Mn) on the accumulation and dynamics of phenolic compounds in olive leaves.

## 2. Materials and Methods

### 2.1. Plant Treatments and Soil Characterization

Before initiating the experiment, plant material was collected from the national collection orchard at the Institute of Agriculture and Tourism in Poreč, Croatia (accession number FRU00268). The experiment was conducted in a greenhouse at the Longo nursery in Rovinj, Croatia (45°4′53″ N; 13°41′30″ E), under natural light and photoperiod and variable ambient temperature and humidity. Average day/night air temperatures were 24/11 °C, and relative humidity ranged from 30 to 70%, as previously described [27]. In brief, self-rooted cuttings of the ‘Leccio del Corno’ olive cultivar were transplanted into 3.6 L pots containing terra rossa soil (Rhodic Cambisol, IUSS Working Group WRB, 2015), supplemented with peat and fertilizer (Plantacote Top K 6M^®^ 10:9:19, Aglukon Spezialdünger GmbH, Düsseldorf, Germany) at 1 kg m^−3^. The growing substrate had a pH (H_2_O) of 6.6, indicating a neutral reaction (HRN ISO 10390:2004) [27,28].

Treatments were applied to 12-month-old olive plantlets as a single foliar application at the beginning of the experiment. Zn, Cu, and Mn treatments were prepared using commercial fertilizers (Tradecorp^®^, Tradecorp International Universal S.A.U., Madrid, Spain), which supply zinc, copper, and manganese in chelated forms to ensure optimal absorption. Stock solutions were prepared by dissolving each product in deionized water to a final concentration of 3 g/L, following the manufacturer’s recommendations for olive cultivation. The corresponding micronutrient concentrations were calculated from their elemental composition.

During the experiment, each plant received 200 mL of tap water twice a week, and sampling was conducted at 30, 60, and 90 days after treatment. The foliar treatments were applied by foliar spraying until runoff, as follows:

- Control treatment (C): water;
- Zn treatment: 6.42 mM, corresponding to 3 g Tradecorp Zn L^−1^;
- Cu treatment: 6.85 mM, corresponding to 3 g Tradecorp Cu L^−1^;
- Mn treatment: 7.1 mM, corresponding to 3 g Tradecorp Mn L^−1^.

### 2.2. Preparation of Plant Material

Treated old olive leaves (OOL) were collected from the middle parts of three plants per sampling for chemical analyses at 30, 60, and 90 days after treatment (DAT). Young olive leaves (YOL)—newly formed, fully expanded leaves that developed after the treatment was applied and were therefore not sprayed—were collected only at the end of the experiment (90 DAT). Ten plants were assigned to each treatment. Sampling was destructive, with three independent plants collected at each of the three sampling times, while one additional plant was maintained as a reserve. Each sampled plant represented one biological replicate (n = 3 per sampling time). Samples collected at each processing time were transferred to the laboratory and rinsed with tap water. Olive leaves were then successively immersed in deionized water with 1% acetic acid and in deionized water and were drained in a perforated vessel for 5 min [29]. Finally, samples were air-dried until reaching a constant weight.

### 2.3. Mineral Concentration Analysis

Dried olive leaves were finely ground (0.5 g), weighed in porcelain crucibles, and dry-ashed at 550 °C for 8 h. The ash was dissolved in 5 mL of 0.6 M hydrochloric acid (Normapur, VWR International, Radnor, PA, USA) at 60 °C for 15 min. The filtrate was collected on Munsell No. 388 filter paper, transferred to PE-graduated tubes, and diluted to a final volume of 50 mL with deionized water [30].

Analyses of minerals (Cu, Mn, and Zn) were conducted using an ICP-MS NexION 300× (PerkinElmer Instruments, Waltham, MA, USA) fitted with an S10 autosampler. A multi-element solution spanning a broad range of element masses was used for instrument tuning (NexION Setup Solution, PerkinElmer, Waltham, MA, USA). Each calibration curve was obtained by preparing at least six ICP-MS standards through appropriate dilutions of the multi-element standard solution with the same acid matrix. Calibration curves with R2 ≥ 0.999 were accepted for concentration calculation, and their ranges were suitable for the investigated analytes. The mean of five runs was obtained for each sample/element. Reagent blanks were prepared and determined in the same way as the samples.

### 2.4. Analysis of Phenolic Compounds

Phenolic compound extraction from olive leaves subjected to different treatments was carried out following Marinova et al. [31], with slight modifications. In brief, 500 mg of ground leaves was extracted with 25 mL of methanol/water (80:20, *v*/*v*), sonicated for 20 min in an ultrasonic bath, and then centrifuged for 10 min at 7000 rpm. The hydrophilic fraction was collected and filtered (through a 0.45 μm pore cellulose acetate filter) for subsequent analysis.

Total phenol content was measured using the modified Folin–Ciocalteu method [32]. The reaction was carried out by mixing the olive leaf extract (250 μL) with deionized water (15 mL) and Folin–Ciocalteu solution (Sigma-Aldrich, Steinheim, Germany). Three minutes later, 2.5 mL of sodium carbonate solution was added to neutralize the reaction, and the flask was filled to volume with deionized water. The mixture was incubated for 90 min at ambient temperature in the dark. Total phenolic absorbance was measured at 725 nm (Perkin-Elmer UV/VIS Lambda Bio 40 spectrophotometer, PerkinElmer, Waltham, MA, USA), and the content was determined using a calibration curve prepared with pure caffeic acid (Sigma-Aldrich, Steinheim, Germany). The results were expressed as mg of caffeic acid equivalents per 100 g of dry matter.

Targeted phenolic compounds were analyzed following the method described in IOC (2017) [33]. High-performance liquid chromatography (HPLC) was performed on a PerkinElmer Series 200 HPLC system (Waltham, MA, USA) equipped with an autosampler, binary pump, vacuum degasser, column oven, and UV/Vis detector. Phenolic compounds were separated on an Ultra Aqueous C18 column (5 μm, 150 × 4.60 mm; Restek, Bellefonte, PA, USA) using a mobile phase consisting of 0.2% phosphoric acid (solvent A) and a methanol/acetonitrile mixture (1:1, *v*/*v*) as solvent B. Chromatography was carried out using the following elution program: the mobile phase initially consisted of 96% solvent A and 4% solvent B, and solvent A was gradually decreased to 50% over 40 min. Subsequently, solvent A was further reduced to 40% over the next 5 min, then to 0% by 60 min. This composition (100% solvent B) was maintained for 8 min. Thereafter, solvent A was linearly increased back to 96% from 68 to 70 min, and this initial mobile phase composition was held for an additional 10 min to allow column re-equilibration. The column temperature was set to 25 °C, and the mobile phase flow rate was maintained at 0.8 mL min^−1^. An injection volume of 20 µL was used, and the absorbance of the phenolic compounds was recorded at 280 nm.

Phenolic compounds were identified by comparing their retention times with those of the corresponding pure standards. The oleuropein calibration curve was used to quantify its derivatives, including dicarboxymethyl oleuropein aglycon and oleuropein aglycon. Phenolic compound concentrations were expressed as mg per 100 g dry weight (DW). All analytical-grade standards and solvents were purchased from Sigma-Aldrich (Steinheim, Germany) and Merck (Darmstadt, Germany). Deionized water (Milli-Q) was used throughout the analyses. For statistical evaluation, individual compounds were grouped into four classes: simple phenols (hydroxytyrosol and tyrosol), secoiridoids (dicarboxymethyl oleuropein aglycone, oleuropein aglycone, and oleuropein), flavonoids (luteolin and apigenin) and phenolic acids (3,4-dihydroxybenzoic, 4-hydroxybenzoic, caffeic, p-coumaric, trans-ferulic and cinnamic acid). Class concentrations were calculated as the sum of the individual compounds within each class.

### 2.5. Statistical Analysis

The experiment was set up in a completely randomized design (n = 3 biological replicates per treatment × sampling time). Data were tested for normality (Shapiro–Wilk) and homogeneity of variance (Levene’s test), transformed when necessary, and then subjected to analysis of variance (ANOVA). Data for old olive leaves were subjected to two-way ANOVA with treatment (C, Cu, Mn, Zn) and sampling time (30, 60, 90 DAT) as fixed factors and their interaction; data for young olive leaves, sampled at a single time point (90 DAT), were subjected to one-way ANOVA with treatment as the only factor. Means were separated by Tukey’s HSD test at *p* < 0.05. When the treatment × sampling time interaction was significant, only the interaction means were compared, and post hoc letters were therefore not assigned to the corresponding main effects. Pearson correlation analyses were performed using all old leaf samples across sampling times (OOL; n = 36), while separate analyses at 90 DAT were conducted for old leaves (OOL; n = 12) and young leaves (YOL; n = 12). The analyses were performed using IBM SPSS Statistics for Windows, Version 21.0 (IBM Corp., Armonk, NY, USA) and TIBCO Statistica version 14.1.0.8 (TIBCO StatSoft, Palo Alto, CA, USA). The heatmap, dendrogram, and supervised PLS-DA were generated using MetaboAnalyst 6.0 Xia Lab, McGill University, Montreal, QC, Canada).

## 3. Results

### 3.1. Nutrient Concentrations in Olive Leaves

The effects of foliar applications of Cu, Mn, and Zn on OOL were evaluated, and their temporal variation was also assessed. Results showed that foliar application significantly increased their accumulation in OOL, reaching high levels (Table 1).

After Mn foliar application, the main effect of the treatment was an increase in Mn (47.94 mg/kg DW) compared with the other treatments. A significantly higher Mn content was recorded at 30 DAT than at 90 DAT.

The interaction between sampling period and treatment showed that Cu foliar fertilization significantly increased Cu levels at 30 DAT (28.77 mg/kg DW) and 60 DAT (22.97 mg/kg DW) compared with the other treatments in the same sampling period (*p* ≤ 0.05). For Zn foliar application, a significant interaction between treatment and duration was observed (*p* ≤ 0.001). Zinc-treated plants had higher Zn levels across all treatments at 60 and 90 DAT, at 29.5 and 29.9 mg/kg DW, respectively (Table 1).

### 3.2. Phenolic Concentrations in Olive Leaves

#### 3.2.1. Total and Simple Phenols

The applied Zn and Mn foliar treatments significantly reduced hydroxytyrosol concentrations compared with the control, regardless of sampling time. Considering the sampling time effect, the hydroxytyrosol concentration was significantly higher at 30 (53.86 mg/100 g DW) and 60 (53.09 mg/100 g DW) DAT, whereas a lower concentration was generally observed at 90 DAT (37.73 mg/100 g DW) (Table 2).

In OOL at 60 DAT, the control treatment showed a higher total phenol concentration (TPC) than all other treatments, but this difference was not observed at 30 or 90 DAT (Table 2). In YOL, the TPC was significantly higher in the leaves from the trees under the Zn treatment (9917.56 mg/100 g DW) than under the other treatments. Copper and Mn applications also resulted in increased TPC levels (8748 mg/100 g DW and 8435.81 mg/100 g DW, respectively) but only when compared to the control (7096.88 mg/100 g DW) (Table 3).

#### 3.2.2. Secoiridoids and Flavonoids

Interactions between Cu, Mn, and Zn application and sampling time (DAT) were significant for measured flavonoids and secoiridoids in old olive leaves (Table 4). The highest decarboxylmethyl oleuropein content was observed in control plants at 30 (195.27 mg/100 g DW) and 60 (218.23 mg/100 g DW) DAT, compared with most other interactions, except Zn × 30. For oleuropein aglycon, the highest content was recorded in Zn-treated plants (162.45 mg/100 g DW) compared with the control at 90 DAT. The oleuropein content of the old leaves was highest in Cu-treated plants at 30 DAT (302.36 mg/100 g DW), but it was not significantly different from C × 30. No significant differences in luteolin were observed among treatments within the same sampling time, whereas apigenin was significantly higher in Zn- than in Cu-treated leaves at 30 DAT (25.84 vs. 11.36 mg/100 g DW).

In young leaves, at 90 DAT, the highest oleuropein content (5104.92 mg/100 g DW) was recorded in leaves from the olives treated with Cu, followed by those treated with Zn (4347.07 mg/100 g DW). Conversely, the lowest levels were observed in leaves treated with Mn and in the control leaves. Furthermore, the Zn treatment showed the highest values for dicarboxymethyl oleuropein (813.72 mg/100 g DW) and oleuropein aglycone (824.57 mg/100 g DW) (Table 3).

#### 3.2.3. Phenolic Acids

The effects of micronutrients on phenolic acids in olive leaves are presented in Table 5. There was no significant effect of Cu, Mn, and Zn application on 4-hydroxybenzoic acid, trans-ferulic acid, and cinnamic acid in OOL. Caffeic acid (5.53 mg/100 g DW) and p-coumaric acid (47.29 mg/100 g DW) contents in OOL were affected, with higher levels in control plants compared with Zn- and Mn-treated plants. Phenolic acids also exhibited distinct profiles across sampling times. The contents of 4-hydroxybenzoic acid (7.2 mg/100 g DW) and caffeic acid (5.87 mg/100 g DW) were higher at 30 DAT, whereas trans-ferulic acid (17.06 mg/100 g DW) had significantly higher concentrations at 90 DAT than at the other sampling times.

The highest 3,4-dihydroxybenzoic acid content was recorded in Zn-treated leaves at 30 DAT (10.75 mg/100 g DW), which did not differ significantly from the remaining 30 DAT or from the control at 60 DAT. There was no significant difference in cinnamic acid concentration among treatments at the same sampling time.

### 3.3. Hierarchical Clustering Analysis (HCA) and Partial Least Squares Discriminant Analysis (PLS-DA)

**Figure 1:**
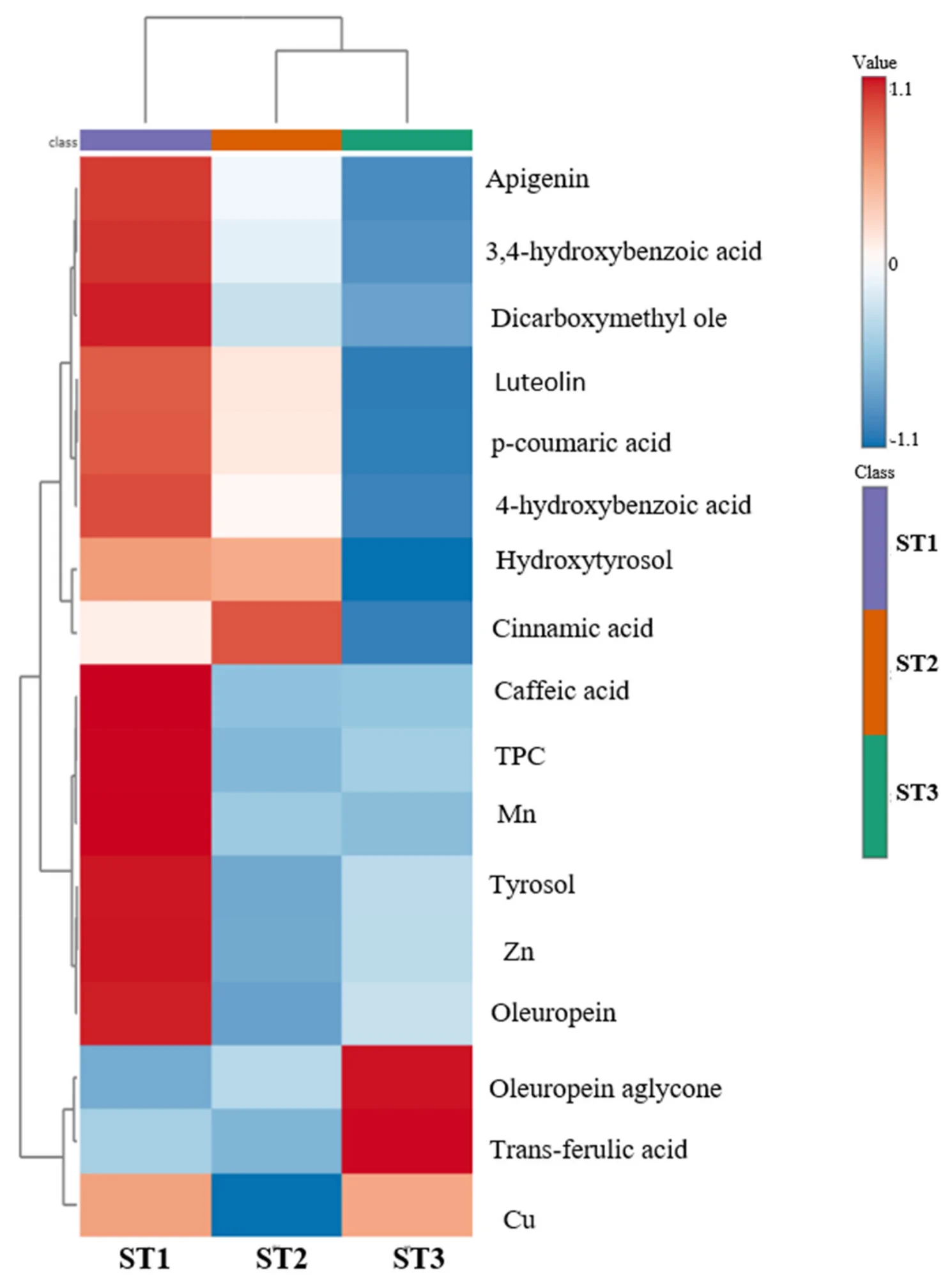
Heatmap combined with hierarchical clustering analysis (HCA) illustrating the relative abundance of phenolic compounds and micronutrients (Cu, Mn and Zn) in old olive leaves. Rows represent the quantified metabolites and micronutrients, while columns correspond to the three sampling times (ST1: 30 DAT, ST2: 60 DAT and ST3: 90 DAT). The color scale represents the relative abundance of each variable, with red indicating higher than average levels and blue indicating lower than average levels. The dendrograms group samples and variables according to similarities in their phenolic profiles, highlighting temporal changes in metabolite composition following foliar fertilization.

To evaluate global variation among samples, a comprehensive cluster analysis of the dataset, based on quantified metabolites and mineral nutrients, was performed. Hierarchical clustering analysis (HCA) was used to classify samples by sampling period. The heatmap analysis, using Ward’s method, grouped the olive leaf samples into three distinct clusters (Figure 1).

For old leaves, the groups were classified by sampling time: ST1 (30 DAT), ST2 (60 DAT), and ST3 (90 DAT) (Figure 1). The first group (ST1) was characterized by the abundance of most of the studied phenolic compounds. The second group (ST2) was characterized by cinnamic acid, followed by hydroxytyrosol. The third group (ST3) was particularly abundant in oleuropein aglycone, trans-ferulic acid, and Cu.

**Figure 2:**
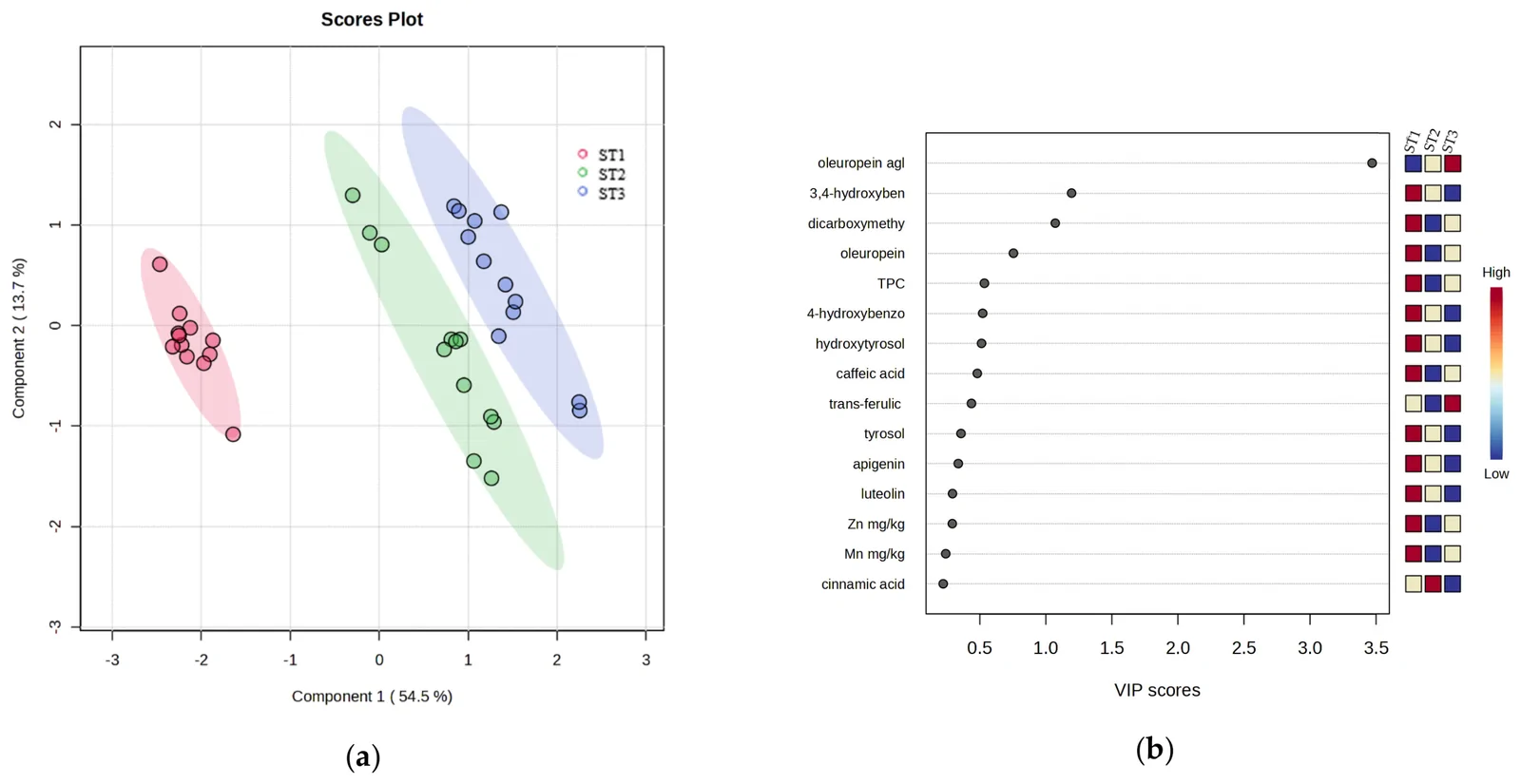
Partial least squares discriminant analysis (PLS-DA) and VIP scores of the phenolic profiles of old olive leaves. The score plot illustrates the discrimination among sampling times according to their metabolite composition. PC1 and PC2 explained 54.5% and 13.7% of the total variance, respectively (**a**). Variables with VIP scores >1 were considered the principal metabolites contributing to group discrimination. ST1, ST2 and ST3 correspond to samples collected at 30, 60 and 90 DAT, respectively (**b**).

Partial least squares discriminant analysis (PLS-DA) was performed to evaluate the discrimination among olive leaf samples subjected to different treatments at different sampling times (Figure 2). For old olive leaves, our results revealed a clear separation between sampling periods for foliar-treated plants. Notably, the 30 DAT samples formed a distinct cluster, separate from the 60 and 90 DAT.

The discriminative power of mineral nutrients (Cu, Zn, and Mn) and phenolic compound parameters was evaluated using their variable importance in projection (VIP) scores (Figure 2). The most discriminative parameter was oleuropein aglycone, with VIP scores of approximately 3.5, followed by 3,4-hydroxybenzoic acid and dicarboxymethyl (VIP scores > 1).

### 3.4. Correlation Analysis

Pearson correlation analysis revealed several significant relationships between mineral concentrations and classes of phenolic compounds in old (at 30, 60 and 90 DAT) and young (at 90 DAT) olive leaves. Overall, in OOL, Cu concentration was positively correlated with secoiridoids (r = 0.569, *p* < 0.001; Table S1). Strong positive correlations were also observed among simple phenols, flavonoids, and phenolic acids (r = 0.616, *p* < 0.001; r = 0.738, *p* < 0.001; r = 0.792, *p* < 0.001, respectively). Conversely, secoiridoids were negatively correlated with simple phenols (r = −0.422, *p* = 0.010) and flavonoids (r = −0.407, *p* = 0.014).

At 90 DAT in OOL, Zn concentration was strongly positively correlated with secoiridoids (r = 0.740, *p* = 0.006; Table S2), whereas Mn concentration showed a strong negative correlation with flavonoids (r = −0.608, *p* = 0.036). Strong positive correlations were also observed between simple phenols and flavonoids (r = 0.725, *p* = 0.008) and between simple phenols and phenolic acids (r = 0.631, *p* = 0.028). A very strong correlation was observed between flavonoids and phenolic acids (r = 0.836, *p* = 0.001).

At 90 DAT in YOL, simple phenols were very strongly positively correlated with flavonoids (r = 0.853, *p* < 0.001, Table S3). Simple phenols and phenolic acids were strongly negatively correlated with secoiridoids (r = −0.710, *p* = 0.010 and r = −0.880, *p* < 0.001, respectively). Copper concentration was strongly positively correlated with flavonoids (r = 0.649, *p* = 0.022), whereas Mn concentration was strongly negatively correlated with flavonoids (r = −0.626, *p* = 0.029). Among mineral elements, Zn and Cu showed a very strong positive correlation (r = 0.857, *p* < 0.001), whereas Zn and Mn (r = −0.886, *p* < 0.001) and Cu and Mn (r = −0.833, *p* = 0.001) showed very strong negative correlations.

## 4. Discussion

Foliar application of Zn, Cu, and Mn affected mineral status and phenolic accumulation in olive leaves, but the response varied across leaf developmental stages and sampling times. These findings indicate that both micronutrient type and leaf age should be considered when evaluating the potential of foliar fertilization to modify olive leaf phenolic profiles.

Importantly, measured Zn, Cu, and Mn concentrations in OOL remained above the critical deficiency levels reported in the literature (Table 1). Zn concentrations in OOL exceeded 13 mg kg^−1^ DW across all control × DAT combinations and under the Zn foliar treatment, indicating that the plants did not experience Zn deficiency. These values were above the commonly reported deficiency threshold for olive leaves, as Souza et al. [15] reported that Zn deficiency in olive trees occurs when foliar Zn concentrations decline to approximately 10 mg kg^−1^ DW. Regarding Cu, Plank et al. [34] reported that Cu deficiency in olive may occur when foliar Cu concentrations decline to around 3 mg kg^−1^ DW. In the present study, Cu concentrations were consistently higher than 11 mg kg^−1^ DW across all control × DAT combinations and under the Cu foliar treatment (Table 1). Concerning Mn, critical levels in plant tissues can vary depending on cultivar, species, and environmental conditions. However, deficiency thresholds are generally reported to range from 1 to 5 mg kg^−1^ DW [35]. In the present study, Mn concentrations were consistently higher than 28 mg kg^−1^ DW across all control × DAT combinations and in the Mn foliar treatment (Table 1). Although all foliar treatments were applied according to the same schedule, the accumulation patterns of Zn, Cu, and Mn in treated and untreated plants differed over time, suggesting element-specific uptake, redistribution, or homeostatic regulation [36,37]. Thus, the delayed increase in Zn concentration at 60 and 90 DAT may result from slower foliar penetration and/or gradual redistribution within the plant, whereas the absence of a significant difference between Zn-treated plants and the control at 30 DAT suggests that the initial response may have been limited or masked by natural variability. Xie et al. [38] noted that a relatively high trichome density may increase leaf hydrophobicity, which may affect foliar fertilization efficiency in olive, as the abaxial surface of olive leaves is characterized by high trichome density [39]. In contrast, as observed in our previous research [17], Mn showed a consistent response throughout the sampling period, whereas the effect of Cu treatment on Cu concentration in OOL was temporary, suggesting stronger physiological regulation and reduced persistence of the initial treatment effect [37].

The results of our experiment showed that TPC, simple phenols, secoiridoids, flavonoids, and phenolic acids in OOL were not consistently affected by the selected foliar micronutrient treatments (Cu, Zn, and Mn) (Table 2, Table 4 and Table 5). Moreover, in OOL, oleuropein was not significantly enhanced by the selected foliar applications, whereas YOL showed a different pattern with significantly higher levels in leaves from the trees under Zn and Cu fertilization. On the other hand, Zn foliar fertilization was associated with higher oleuropein aglycone levels at 90 DAT in both OOL and YOL (Table 3 and Table 4). Previous studies have shown that zinc fertilizers can increase plant phenolic compounds [40,41], and Zn application has also been associated with phenolic acid accumulation by stimulating key enzymes, including phenylalanine ammonia-lyase (PAL) and cinnamate-4-hydroxylase (C4H), and by upregulating genes involved in phenolic acid biosynthesis [42,43,44]. However, enzyme activities and gene expression were not assessed in the present study; therefore, the Zn-associated changes observed here should be interpreted as changes in phenolic accumulation rather than as direct evidence of pathway regulation.

It has also been shown that copper deficiency can decrease phenolic content. This nutrient plays an important role in the shikimic acid pathway and the biosynthesis of phenolic compounds [45,46,47]. Previous studies have reported that oleuropein represents one of the main secoiridoid phenolic compounds in olive tissues and that its degradation may lead to the formation of aglycone forms and related derivatives, which may subsequently be transformed into phenolic acids such as 3,4-dihydroxybenzoic acid [48,49]. However, there was no significant impact of Zn treatment on 3,4-dihydroxybenzoic acid in OOL. Although these compounds may be metabolically related, their concentrations do not necessarily correlate significantly, suggesting that their accumulation may reflect distinct metabolic dynamics or physiological responses [50].

Several studies indicate that higher TPC levels are associated with mineral fertilization treatments, such as B or Mn [18,27]. This result is consistent with our findings only in YOL, where we observed an increase in TPC across all micronutrient treatments. Because young leaves were sampled only at the end of the experiment (at 90 DAT), this response should be interpreted primarily as a developmental-stage effect rather than a direct time-dependent response. Young leaves are generally characterized by more active secondary metabolism and stronger antioxidant capacity, which may explain their higher responsiveness to micronutrient application. It has been demonstrated that phenolic compound levels, especially secoiridoids, are higher and accumulate to greater levels in young leaves than in mature leaves [16,51]. Pasković et al. [27] also reported that young leaves of plants treated with boron fertilization are rich in phenols, particularly secoiridoids. This trend has been observed in olive trees and other plant species, possibly due to more intense metabolic activity and greater stimulation of the phenylpropanoid pathway [52]. This response is generally more pronounced in YOL, where defense mechanisms are more active [53].

However, in old olive leaves, the response differed. Only at 60 DAT did Mn-, Cu-, and Zn-treated leaves show lower TPC values than the control, indicating that micronutrient application may not uniformly stimulate phenolic accumulation in mature tissues. This reduction may be partly explained by the role of phenolic compounds in regulating metal-related stress. Phenolics can interact with metal ions, potentially modulating metal reactivity and oxidative stress responses [54,55,56]. Similar fertilizer-associated reductions in total polyphenol content have also been reported in roselle (*Hibiscus sabdariffa*) leaves, although the effect was described as non-significant [57].

In OOL, hierarchical clustering analysis revealed a clear temporal structure in phenolic profiles, with ST1 generally showing higher abundance across most phenolic classes, whereas later sampling times exhibited more specific metabolite patterns, particularly cinnamic acid and hydroxytyrosol in ST2 and oleuropein aglycone and trans-ferulic acid in ST3 (Figure 1). This is consistent with previous research showing that olive leaf phenolic profiles are strongly influenced by sampling time and cultivar-related factors [58]. The separation of secoiridoids from several simple phenols and flavonoids suggests that these phenolic groups may follow partially distinct accumulation dynamics during leaf maturation. This interpretation was further supported by correlation analysis, which indicated that leaf micronutrient status was associated with the distribution of phenolic classes in both OOL and YOL (Tables S1–S3). In OOL, Cu and Zn were mainly associated with secoiridoid variation, whereas in YOL, Cu was more closely related to flavonoid accumulation. Conversely, in YOL, Mn showed a negative association with flavonoids, suggesting a different pattern of phenolic modulation compared with Cu and Zn. However, these associations should not be interpreted as direct evidence of causal stimulation, metabolic competition, or pathway regulation. Previous studies have shown that Mn, Zn, and Cu nutrition can influence phenolic metabolism in olive and other plant systems; however, the direction and magnitude of these responses appear to depend on plant species or genotype, tissue type or developmental stage, treatment concentration, and exposure time [17,26,51,58,59,60,61,62,63,64].

It remains unclear whether the observed changes in phenolic metabolism were due to improved micronutrient nutrition or to mild metal-induced oxidative stress, as oxidative stress markers and key phenylpropanoid enzymes were not measured. Another limitation is that each micronutrient was applied at only one commercially recommended rate (3 g L^−1^), without testing different doses. Although leaf micronutrient concentrations remained above the deficiency thresholds reported for olive (Table 1), suggesting that the responses were not due to the correction of a deficiency, dose–response experiments, combined with measurements of oxidative stress and enzyme activity, are needed to distinguish nutritional from stress-related effects on phenolic metabolism.

## 5. Conclusions

In a context where olive leaves are increasingly considered a sustainable source of high-value bioactive compounds, controlling agronomic factors that can modulate their phenolic composition represents a major scientific and industrial challenge.

The results of this study show that foliar fertilization with micronutrients (Zn, Cu, and Mn) is a promising means of influencing the phenolic profile of leaves, depending on the sampling time and developmental stage. In old leaves, secoiridoid concentrations were highest at 30 DAT, whereas at the later sampling times, the phenolic profile was progressively reorganized: total phenols, hydroxytyrosol, dicarboxymethyl oleuropein, oleuropein and several phenolic acids decreased, while oleuropein aglycone and trans-ferulic acid increased. Flavonoid concentrations were not significantly affected by sampling time. Furthermore, young leaves accumulated total phenols and secoiridoids at concentrations several times those measured in mature leaves at the same sampling date. Correlation analyses also revealed specific modulation of the different phenolic classes by micronutrients, with Cu and Zn associated with secoiridoids, while Mn was associated with a decrease in flavonoids.

Although the molecular mechanisms involved were not investigated in this work, the results obtained highlight a temporal modulation of the phenolic profile in response to foliar fertilization. Further studies, including multiple micronutrient doses, markers of oxidative stress, enzymatic activities, and transcriptomic and metabolomic approaches, are needed to clarify the observed micronutrient-associated changes and elucidate the underlying regulatory mechanisms. Overall, combining targeted foliar micronutrient application with harvesting of young olive leaves may be a promising phytochemical farming approach for producing phenolic-rich plant material suitable for nutraceutical use and product development.

## Figures and Tables

**Table 1:** The effect of foliar-applied nutrients (6.42 mM Zn, 6.85 mM Cu, and 7.1 mM Mn) and sampling time (30, 60 and 90 DAT) on nutrient concentrations in old olive leaves.

Source of Variation	Zinc	Copper	Manganese
	(mg/kg DW)		
Control (C)	18.53 ± 7.16	15.31 ± 3.68	32.37 ± 6.54 b
Cu	18.78 ± 3.92	24.61 ± 4.31	36.36 ± 3.92 b
Mn	16.86 ± 1.86	15.21 ± 3.53	47.94 ± 7.79 a
Zn	29.11 ± 2.82	17.27 ± 5.39	32.01 ± 5.45 b
**DAT**			
30	23.92 ± 5.48	19.81 ± 5.95	40.98 ± 9.54 a
60	18.73 ± 6.6	14.76 ± 5.28	35.44 ± 8.98 ab
90	19.81 ± 6.6	19.73 ± 4.51	35.08 ± 6.97 b
**Treatment × DAT**			
C × 30	27.7 ± 3.35 a	18.53 ± 1.78 bcd	35.97 ± 7.56
C × 60	14.33 ± 1.04 c	11.17 ± 0.78 d	28.93 ± 7.08
C × 90	13.57 ± 1.81 c	16.23 ± 2.8 bcd	32.2 ± 5.14
Cu × 30	22.2 ± 5.45 ab	28.77 ± 4.43 a	37.37 ± 5.88
Cu × 60	16.1 ± 1.08 bc	22.97 ± 1.96 ab	34.97 ± 3.73
Cu × 90	18.03 ± 1.19 bc	22.1 ± 3.4 abc	36.73 ± 2.87
Mn × 30	17.83 ± 1.81 bc	15.6 ± 1.73 bcd	54.1 ± 7.48
Mn × 60	15 ± 1.5 bc	11.37 ± 2.37 d	47.37 ± 6.14
Mn × 90	17.73 ± 0.74 bc	18.67 ± 0.92 bcd	42.37 ± 6.69
Zn × 30	27.93 ± 3.96 a	16.33 ± 1.3 bcd	36.5 ± 2.72
Zn × 60	29.5 ± 0.95 a	13.53 ± 1.88 cd	30.5 ± 5.27
Zn × 90	29.9 ± 3.47 a	21.93 ± 7.48 abc	29.03 ± 6.05
**Treatment**	***	***	***
**DAT**	***	***	*
**T × DAT**	***	*	ns

Values are means ± SD (n = 3). Treatment, DAT, and their interaction were evaluated by two-way ANOVA followed by Tukey’s HSD test (*p* < 0.05). Different lowercase letters indicate significant differences within columns. ns, not significant; * *p* < 0.05; *** *p* < 0.001. For significant treatment × DAT interactions, letters are shown only for interaction means and not for the corresponding main effects.

**Table 2:** The effect of foliar-applied nutrients (6.42 mM Zn, 6.85 mM Cu and 7.1 mM Mn) and sampling time (30, 60, and 90 DAT) on total and simple phenol concentrations in old olive leaves.

Source of Variation	Total Phenols	Simple Phenols	
	TPC	Hydroxytyrosol	Tyrosol
	(mg/100 g DW)		
**Treatment**			
Control (C)	1988.19 ± 438.07	65.59 ± 25.49 a	13.52 ± 3.97
Cu	1429.11 ± 361.81	46.96 ± 11.57 ab	15.14 ± 10.39
Mn	1519.39 ± 728.13	42.65 ± 15.6 b	10.32 ± 3.68
Zn	1367.17 ± 615.77	37.77 ± 13.24 b	10.78 ± 4.27
**DAT**			
30	2075.15 ± 413.8	53.86 ± 10.53 a	13.76 ± 2.8
60	1289.29 ± 610.38	53.09 ± 25.8 a	11.55 ± 4.03
90	1363.46 ± 369.51	37.73 ± 16.68 b	12.02 ± 9.95
**Treatment × DAT**			
C × 30	2187.58 ± 190.4 abc	54.1 ± 9.50	13.65 ± 2.82
C × 60	2250.67 ± 238.03 ab	88.33 ± 27.98	16.57 ± 4.72
C × 90	1526.33 ± 437.93 cde	54.16 ± 23.56	10.35 ± 1.98
Cu × 30	1517.25 ± 406.28 cde	55.09 ± 11.35	12.34 ± 3.51
Cu × 60	1055.25 ± 80.62 de	47.155 ± 5.7	11.31 ± 1.32
Cu × 90	1714.83 ± 91.04 bcd	38.63 ± 13.08	21.77 ± 17.84
Mn × 30	2442.42 ± 189.09 a	57.95 ± 17.24	14.03 ± 3.82
Mn × 60	843.42 ± 36.99 e	41.7 ± 3.28	9.42 ± 1.53
Mn × 90	1272.33 ± 169.17 de	28.3 ± 2.18	7.53 ± 1.94
Zn × 30	2153.33 ± 85.77 abc	48.3 ± 4.40	15.01 ± 1.64
Zn × 60	1007.83 ± 314.68 e	35.19 ± 15.65	8.9 ± 2.81
Zn × 90	940.33 ± 127.57 e	29.83 ± 12.89	8.43 ± 4.7
**Treatment**	***	**	ns
**DAT**	***	*	ns
**T × DAT**	***	ns	ns

Values are means ± SD (n = 3). Treatment, DAT, and their interaction were evaluated by two-way ANOVA followed by Tukey’s HSD test (*p* < 0.05). Different lowercase letters indicate significant differences within columns. ns, not significant; * *p* < 0.05; ** *p* < 0.01; *** *p* < 0.001. For significant treatment × DAT interactions, letters are shown only for interaction means and not for the corresponding main effects.

**Table 3:** The effect of foliar-applied nutrients (6.42 mM Zn, 6.85 mM Cu, and 7.1 mM Mn) on secoiridoids and total phenol concentrations (mg/100 g DW) in young olive leaves at 90 DAT.

Source of Variation		Secoiridoids		
	Total Phenols (TPC)	Dicarboxymethyl Ole	Oleuropein Aglycone	Oleuropein
	(mg/100 g DW)			
**Treatment**				
Control (C)	7096.88 ± 300.38 c	544.81 ± 13.67 b	548.29 ± 45.35 c	2661.19 ± 184.97 c
Cu	8748 ± 289.5 b	539.45 ± 16.36 b	713.13 ± 71.16 b	5104.92 ± 267.71 a
Mn	8435.81 ± 276.57 b	598.02 ± 55.84 b	555.9 ± 53.25 c	2965.45 ± 226.2 c
Zn	9917.56 ± 556.57 a	813.72 ± 77.61 a	824.57 ± 0.11 a	4347.07 ± 8.03 b
**Treatment**	***	***	***	***

Values are means ± SD (n = 3). Treatment, DAT, and their interaction were evaluated by two-way ANOVA followed by Tukey’s HSD test (*p* < 0.05). Different lowercase letters indicate significant differences within columns. ns, not significant; *** *p* < 0.001.

**Table 4:** The effect of foliar-applied nutrients (6.42 mM Zn, 6.85 mM Cu, and 7.1 mM Mn) and sampling time (30, 60, and 90 DAT) on secoiridoid and flavonoid concentrations in old olive leaves.

Source of Variation		Secoiridoids		Flavonoids	
	Dicarboxymethyl Ole	Oleuropein Aglycone	Oleuropein	Luteolin	Apigenin
(mg/100 g DW)					
**Treatment**					
Control (C)	168.63 ± 73.23	33.07 ± 36.36	115.87 ± 76.08	55.25 ± 18.35	16.12 ± 4.75
Cu	55.28 ± 43.13	26.08 ± 26.62	162.29 ± 123.51	35.48 ± 10.35	15.01 ± 4.6
Mn	47.27 ± 48.25	26.92 ± 29.36	116.04 ± 66.54	33.66 ± 10.94	12.77 ± 5.43
Zn	59.88 ± 65.39	58.48 ± 78.26	81.92 ± 69.29	47.3 ± 22.15	18.78 ± 7.98
**DAT**					
30	140.16 ± 18.88	0 ± 0	209.43 ± 78.39	47.38 ± 14.98	17.57 ± 6.92
60	66.86 ± 91.85	17.26 ± 3.2	53.13 ± 35.79	43.68 ± 18.73	15.53 ± 3.39
90	41.29 ± 32.54	91.15 ± 44.75	94.54 ± 54.39	37.71 ± 20.12	13.9 ± 6.94
**Treatment × DAT**					
C × 30	195.27 ± 87.14 a	0 ± 0 e	209.06 ± 25.33 ab	55.17 ± 14.98 ab	17.57 ± 5.25 ab
C × 60	218.23 ± 18.88 a	19.37 ± 0.54 d	93.62 ± 33.51 bcd	66.35 ± 18.73 a	17.92 ± 3.1 ab
C × 90	92.39 ± 5.38 bcd	79.83 ± 9.33 b	44.92 ± 8.06 cd	44.24 ± 20.12 ab	12.86 ± 5.42 ab
Cu × 30	111.25 ± 4.33 bc	0 ± 0 e	302.36 ± 95.65 a	28.96 ± 8.11 ab	11.36 ± 2.21 b
Cu × 60	16.42 ± 1.21 de	18.89 ± 0.8 de	64.41 ± 26.89 cd	29.84 ± 5.22 ab	13.27 ± 2.65 ab
Cu × 90	38.16 ± 4.17 cde	59.36 ± 8.67 c	120.11 ± 68.42 bcd	47.64 ± 1.37 ab	20.41 ± 2.06 ab
Mn × 30	109.13 ± 23.37 bc	0 ± 0 e	178.98 ± 50.33 abc	37.63 ± 14.92 ab	15.54 ± 7.75 ab
Mn × 60	9.27 ± 0.23 e	17.79 ± 0.8 de	39.96 ± 6.81 d	39.89 ± 2.93 ab	14.89 ± 0.71 ab
Mn × 90	23.42 ± 2.7 de	62.97 ± 16.95 bc	129.19 ± 15.67 bcd	23.47 ± 3.19 b	7.87 ± 1.77 b
Zn × 30	144.98 ± 25.35 ab	0 ± 0 e	147.3 ± 36.54 bcd	67.75 ± 2.87 a	25.84 ± 2.11 a
Zn × 60	23.48 ± 7.31 de	13 ± 4.08 de	14.53 ± 2.43 d	38.65 ± 2.87 a	16.04 ± 5.38 ab
Zn × 90	11.19 ± 0.49 de	162.45 ± 6.11 a	83.93 ± 68.09 bcd	35.49 ± 28.37 ab	14.47 ± 10.38 ab
**Treatment**	***	***	**	**	ns
**DAT**	***	***	***	ns	ns
**T × DAT**	***	***	*	*	*

Values are means ± SD (n = 3). Treatment, DAT, and their interaction were evaluated by two-way ANOVA followed by Tukey’s HSD test (*p* < 0.05). Different lowercase letters indicate significant differences within columns. ns, not significant; * *p* < 0.05; ** *p* < 0.01; *** *p* < 0.001. For significant treatment × DAT interactions, letters are shown only for interaction means and not for the corresponding main effects.

**Table 5:** The effect of foliar-applied nutrients (6.42 mM Zn, 6.85 mM Cu, and 7.1 mM Mn) and sampling time (30, 60, and 90 DAT) on phenolic acid concentrations in old olive leaves.

Source of Variation	3,4-Hydroxybenzoic Acid	4-Hydroxybenzoic Acid	Caffeic Acid	p-Coumaric Acid	Trans-Ferulic Acid	Cinnamic Acid
(mg/100 g DW)						
**Treatment**						
Control (C)	6.26 ± 2.22	7.14 ± 1.88	5.53 ± 1.95 a	47.29 ± 11.09 a	14.11 ± 5.48	0.55 ± 0.15
Cu	4.84 ± 2.77	6.23 ± 1.72	4.75 ± 1.25 ab	40.52 ± 8.02 ab	13.32 ± 6.57	0.49 ± 0.19
Mn	4.6 ± 2.81	5.78 ± 1.79	3.35 ± 1.51 b	32.54 ± 4.66 b	12.77 ± 3.36	0.49 ± 0.08
Zn	6.01 ± 3.86	5.46 ± 1.73	4.11 ± 2.74 ab	34.1 ± 10.4 b	13.4 ± 6.37	0.46 ± 0.16
**DAT**						
30	8.57 ± 2.23	7.2 ± 1.65 a	5.87 ± 1.99 a	40.17 ± 7.13	11.91 ± 4.38 b	0.5 ± 0.14
60	4.99 ± 1.62	6.2 ± 1.76 ab	3.69 ± 1.92 b	38.85 ± 11	11.22 ± 2.51 b	0.55 ± 0.09
90	2.72 ± 0.94	5.06 ± 1.5 b	3.73 ± 1.45 b	36.81 ± 12.8	17.06 ± 6.63 a	0.44 ± 0.17
**Treatment × DAT**						
C × 30	7.69 ± 1.8 abcd	7.21 ± 1.12	7.12 ± 2.44	41.58 ± 6.37	14.89 ± 3.92	0.65 ± 0.09 a
C × 60	7.41 ± 0.51 abcde	8.59 ± 1.59	4.94 ± 0.6	54.97 ± 7.71	9.53 ± 1.65	0.64 ± 0.06 ab
C × 90	3.69 ± 1.5 ef	5.62 ± 1.94	4.52 ± 1.74	45.31 ± 15.77	17.92 ± 6.93	0.38 ± 0.09 ab
Cu × 30	8.04 ± 2.57 ab	7.48 ± 2.23	5.06 ± 1.24	44.92 ± 6.67	9.17 ± 2.51	0.35 ± 0.04 ab
Cu × 60	3.76 ± 0.3 def	5.74 ± 0.47	5.62 ± 0.55	35.25 ± 1.1	9.24 ± 1.22	0.56 ± 0.13 ab
Cu × 90	2.73 ± 0.4 f	5.48 ± 1.78	3.56 ± 1.01	41.39 ± 11.82	21.54 ± 3.58	0.57 ± 0.27 ab
Mn × 30	7.8 ± 2.31 abc	7.18 ± 2.78	4.13 ± 1.87	33.05 ± 8.57	11.97 ± 5.99	0.46 ± 0.07 ab
Mn × 60	3.87 ± 0.88 cdef	5.3 ± 0.34	2.48 ± 1.83	32.31 ± 0.66	13.02 ± 1.52	0.5 ± 0.065 ab
Mn × 90	2.12 ± 0.37 f	4.85 ± 0.64	3.43 ± 0.44	32.26 ± 3.54	13.32 ± 2.33	0.53 ± 0.11 ab
Zn × 30	10.75 ± 1.59 a	6.92 ± 0.92	7.16 ± 0.65	41.14 ± 2.58	11.62 ± 4.84	0.57 ± 0.14 ab
Zn × 60	4.94 ± 0.61 bcdef	5.19 ± 1.57	1.74 ± 0.73	32.88 ± 8.8	13.09 ± 2.83	0.51 ± 0.1 ab
Zn × 90	2.34 ± 1.06 f	4.27 ± 1.81	3.43 ± 2.45	28.27 ± 14.85	15.49 ± 10.92	0.29 ± 0.06 b
**Treatment**	*	ns	*	**	ns	ns
**DAT**	***	*	**	ns	*	ns
**T × DAT**	*	ns	ns	ns	ns	*

Values are means ± SD (n = 3). Treatment, DAT, and their interaction were evaluated by two-way ANOVA followed by Tukey’s HSD test (*p* < 0.05). Different lowercase letters indicate significant differences within columns. ns, not significant; * *p* < 0.05; ** *p* < 0.01; *** *p* < 0.001. For significant treatment × DAT interactions, letters are shown only for interaction means and not for the corresponding main effects.

## Data Availability

The data supporting our findings and analyses are included in the article itself. Readers can access this data by referring to the article.

## Supplementary Materials

The following supporting information can be downloaded at https://www.mdpi.com/article/10.3390/plants15162503/s1, Table S1. Correlation matrix between micronutrients and phenolic compound classes in old olive leaves, overall days after treatment (OOL); Table S2. Correlation matrix between micronutrients and phenolic compound classes in old olive leaves (OOL) at 90 DAT; Table S3. Correlation matrix between micronutrients and phenolic compound classes in young olive leaves (YOL) at 90 DAT.
