# Supplementary material for: Targeted Foliar Micronutrient Modulates Phenolic Accumulation in Olive Leaves
Source: Plants (Basel). 2026 Aug 19;15(16):2503. doi: 10.3390/plants15162503 (PMC13516400; doi:10.3390/plants15162503)
Supplement: Supplementary file 1 [file plants-15-02503-s001.zip › plants-4451335-supplementary.pdf]

Supplementary material

Table S1. Correlation matrix between micronutrients and phenolic compound classes in old olive leaves, overall days after treatment (OOL)

		Zn	Cu	Mn	Simple phenols	Secoiridoids	Flavonoids	Phenolic acids
Zn	r	1	,203	-,258	-,270	,232	,153	-,034
	P value		,234	,129	,111	,174	,372	,844
Cu	r	,203	1	-,091	-,046	**,569****	-,183	,036
	P value	,234		,596	,789	,000	,286	,836
Mn	r	-,258	-,091	1	-,028	-,164	-,215	-,170
	P value	,129	,596		,871	,340	,208	,323
Simple phenols	r	-,270	-,046	-,028	1	**-,422***	**,616****	**,738****
	P value	,111	,789	,871		,010	,000	,000
Secoiridoids	r	,232	**,569****	-,164	**-,422***	1	**-,407***	-,295
	P value	,174	,000	,340	,010		,014	,081
Flavonoids	r	,153	-,183	-,215	**,616****	**-,407***	1	**,792****
	P value	,372	,286	,208	,000	,014		,000
Phenolic acids	r	-,034	,036	-,170	**,738****	-,295	**,792****	1
	P value	,844	,836	,323	,000	,081	,000	
	N	36	36	36	36	36	36	36

**. The correlation is significant at the 0.01 level

*. The correlation is significant at the 0.05 level

Table S2. Correlation matrix between micronutrients and phenolic compound classes in old olive leaves (OOL) at 90 DAT

		Zn	Cu	Mn	Simple phenols	Secoiridoids	Flavonoids	Phenolic acids
Zn	r	1	,469	-,411	-,074	**,740****	,201	-,012
	P value		,124	,184	,820	,006	,530	,971
Cu	r	,469	1	-,264	,066	,351	,446	,204
	P value	,124		,408	,838	,263	,146	,524
Mn	r	-,411	-,264	1	-,482	-,427	**-,608***	-,534
	P value	,184	,408		,112	,167	,036	,074
Simple phenols	r	-,074	,066	-,482	1	-,233	**,725****	**,631***
	P value	,820	,838	,112		,467	,008	,028
Secoiridoids	r	,740**	,351	-,427	-,233	1	,230	,273
	P value	,006	,263	,167	,467		,471	,390
Flavonoids	r	,201	,446	**-,608***	**,725****	,230	1	**,836****
	P value	,530	,146	,036	,008	,471		,001
Phenolic acids	r	-,012	,204	-,534	**,631***	,273	**,836****	**1**
	P value	,971	,524	,074	,028	,390	,001	
	N	12	12	12	12	12	12	12

**. The correlation is significant at the 0.01 level

*. The correlation is significant at the 0.05 level

Table S3. Correlation matrix between micronutrients and phenolic compound classes in young olive leaves (YOL) at 90 DAT

		Zn	Cu	Mn	Simple phenols	Secoiridoids	Flavonoids	Phenolic acids
Zn	r	1	**,857****	**-,886****	,187	,133	,556	-,394
	P value		,000	,000	,560	,679	,061	,205
Cu	r	,857**	1	**-,833****	,411	,072	**,649***	-,416
	P value	,000		,001	,185	,824	,022	,178
Mn	r	-,886**	-,833**	1	-,457	,274	**-,626***	-,005
	P value	,000	,001		,136	,388	,029	,988
Simple phenols	r	,187	,411	-,457	1	**-,710****	**,853****	,306
	P value	,560	,185	,136		,010	,000	,334
Secoiridoids	r	,133	,072	,274	**-,710****	1	-,447	**-,880****
	P value	,679	,824	,388	,010		,146	,000
Flavonoids	r	,556	**,649***	**-,626***	**,853****	-,447	1	-,020
	P value	,061	,022	,029	,000	,146		,952
Phenolic acids	r	-,394	-,416	-,005	,306	**-,880****	-,020	1
	P value	,205	,178	,988	,334	,000	,952	
	N	12	12	12	12	12	12	12

**. The correlation is significant at the 0.01 level
*. The correlation is significant at the 0.05 level
